# MRD in multiple myeloma: the clinical perspective

**DOI:** 10.3389/fonc.2025.1740112

**Published:** 2026-02-27

**Authors:** Tommaso Caravita di Toritto, Angela Rago

**Affiliations:** UOSD Ematologia, ASL Roma 1, Rome, Italy

**Keywords:** multiple myeloma, minimal residual disease, MRD, progression-free survival, next-generation sequencing, flow cytometry

## Abstract

Minimal residual disease (MRD) has emerged as a key prognostic factor in multiple myeloma (MM), allowing a more accurate evaluation of treatment efficacy beyond conventional complete remission. High-sensitivity techniques, including next-generation flow cytometry (NGF), next-generation sequencing (NGS), and allele-specific oligonucleotide quantitative PCR, enable detection of residual disease at levels of 10^−5^–10^−6^. Achieving MRD negativity is consistently associated with prolonged progression-free survival (PFS) and overall survival (OS) across different disease settings. In 2024, the U.S. Food and Drug Administration Oncologic Drugs Advisory Committee unanimously recognized MRD negativity as a primary endpoint in MM clinical trials, reinforcing its role as a validated surrogate of clinical benefit. This review summarizes current MRD detection methods and discusses how MRD assessment, interpreted in the context of recent pivotal clinical trials, may provide a practical framework to guide future treatment strategies in MM.

## Introduction

Multiple myeloma (MM) is a biologically heterogeneous plasma cell malignancy characterized by repeated relapses despite substantial therapeutic advances (1–4). Although modern frontline regimens allow complete remission (CR) rates exceeding 70%, residual disease often persists below the limits of conventional response criteria, contributing to disease recurrence (5–7).

The concept of minimal residual disease (MRD) was formally incorporated into the International Myeloma Working Group (IMWG) response criteria in 2015, recognizing its superior sensitivity and prognostic value compared with standard CR (7). Since then, MRD negativity has emerged as one of the most powerful predictors of long-term outcomes in MM, across both newly diagnosed and relapsed settings (2, 4, 5).

Beyond its established prognostic significance, increasing evidence from recent clinical trials suggests that MRD assessment may offer a practical tool to support future response-adapted treatment strategies in MM (8–14).

## MRD detection techniques

MRD can be assessed in the bone marrow using multiparametric techniques or evaluated at the whole-body level through functional imaging (15, 16).

Next-generation flow cytometry (NGF) allows the identification of aberrant plasma cells with sensitivities reaching 10^−5^–10^−6^ and does not require a baseline diagnostic sample (15). NGF is widely applicable and standardized through EuroFlow protocols, although its accuracy may be influenced by hemodilution and sampling variability.

Next-generation sequencing (NGS) enables highly sensitive detection of clonal immunoglobulin gene rearrangements, with sensitivities up to 10^−6^ (16). NGS provides reproducible results across centers but requires a baseline sample and centralized laboratory platforms.

Allele-specific oligonucleotide quantitative PCR (ASO-qPCR) can achieve sensitivities around 10^−5^ but is technically demanding and has been largely supplanted by NGS in contemporary clinical trials (17).

Extramedullary disease and spatial heterogeneity can be assessed using imaging techniques. Positron emission tomography/computed tomography (PET/CT) and whole-body magnetic resonance imaging (MRI), particularly when including diffusion-weighted imaging (DWI), complement bone marrow-based MRD evaluation (18, 19). The combination of molecular and imaging negativity defines the deepest category of response (2).

## Clinical relevance of MRD

Across multiple prospective studies and randomized clinical trials, MRD negativity has been consistently associated with improved PFS and OS in both newly diagnosed and relapsed/refractory MM (4, 5). This prognostic value is maintained across treatment settings, including transplant-eligible and transplant-ineligible patients (8–14).

Importantly, MRD negativity retains its predictive significance even in patients with high-risk cytogenetic abnormalities, suggesting that deep responses may partially mitigate adverse biological features (2, 3, 20).

## MRD kinetics

Beyond single timepoint assessment, MRD kinetics provide additional clinically relevant information. Sustained MRD negativity over time has been associated with the most favorable long-term outcomes. Conversely, persistent MRD positivity identifies patients at increased risk of early progression. Conversion from MRD-negative to MRD-positive status frequently precedes biochemical relapse, potentially allowing earlier therapeutic intervention (2, 21).

## MRD-guided treatment strategies: clinical implications

Recent pivotal trials have provided insights into how MRD assessment may inform future treatment strategies.

In transplant-eligible patients, studies such as PERSEUS and MASTER have demonstrated that modern quadruplet induction regimens achieve high rates of MRD negativity (11, 12, 13). In this setting, MRD assessment may help identify patients who derive maximal benefit from consolidation strategies or, conversely, those who may be candidates for simplified maintenance approaches.

In transplant-ineligible patients, trials including MAIA, CEPHEUS, IMROZ, and ISKIA have shown that antibody-based combinations result in deep and durable responses (8–9, 14). In this context, MRD evaluation could support treatment optimization by identifying patients who may benefit from prolonged therapy or intensified approaches.

Following cellular therapies, including CAR-T cells, early MRD negativity has been strongly associated with durable remissions (22). Emerging data also suggest that MRD dynamics may inform the integration and sequencing of novel immunotherapies, such as bispecific antibodies, although further prospective validation is required (23).

## Integration of MRD with imaging

Bone marrow MRD assessment alone may underestimate disease burden due to spatial heterogeneity. Functional imaging with PET/CT or whole-body MRI with DWI provides complementary information by detecting focal or extramedullary disease (18, 19, 23). Combined molecular and imaging MRD negativity identifies patients with the deepest responses and the most favorable outcomes (2).

## Challenges in clinical implementation

Despite strong evidence supporting its prognostic value, routine MRD testing faces challenges, including assay standardization, access to high-sensitivity technologies, cost considerations, and the lack of universally accepted MRD-driven treatment algorithms (2, 6).

## Future perspectives

Ongoing research aims to refine MRD detection techniques, explore non-invasive approaches such as circulating tumor DNA, and prospectively validate MRD-guided treatment strategies. Integrating MRD into risk-adapted, personalized treatment pathways may help optimize long-term disease control while minimizing treatment-related toxicity (2).

## Conclusion

MRD assessment has evolved from a research tool to a key component of MM management. Beyond its prognostic significance, MRD evaluation—interpreted in the context of recent clinical trial data—may support future response-adapted therapeutic strategies. Continued efforts toward standardization and clinical integration will be essential to fully realize the potential of MRD-guided care.
